# Lipid Abnormalities and Electrocardiographic Changes in Patients With Psoriasis in a Tertiary Care Hospital

**DOI:** 10.7759/cureus.82164

**Published:** 2025-04-13

**Authors:** Arthi Rajendran, Manu Vidhya Harikumar, Adikrishnan Swaminathan

**Affiliations:** 1 Dermatology, Sri Ramachandra Institute of Higher Education and Research, Chennai, IND

**Keywords:** cardiovascular disease, dyslipidemia, ecg, pasi, psoriasis

## Abstract

Background

Psoriasis is a chronic, immune-mediated inflammatory disease primarily affecting the skin and joints, but it also induces systemic inflammation that disrupts metabolic processes. This systemic involvement contributes to a myriad of comorbidities, including diabetes, dyslipidemia, hypertension, obesity, cardiovascular disease, and psoriatic arthritis.

Objective

This study aimed to examine the relationship between psoriasis and the development of dyslipidemia and, ultimately, cardiovascular diseases.

Materials and methods

A comparative cross-sectional study was conducted with a total of 164 individuals (82 psoriasis cases and 82 controls) within a study period of two years (June 2022 to June 2024). The severity of psoriasis was calculated using the psoriasis area and severity index (PASI) score. Fasting lipid levels and electrocardiography (ECG) were done for patients who consented.

Results

Among 82 cases, the mean PASI score was 6.89, with the majority (37, 45.1%) having mild psoriasis. The prevalence of dyslipidemia was significantly higher in psoriasis patients (61, 74.3%) compared to controls (34, 41.4%), with the majority (26, 31.71%) having elevated low-density lipoprotein (LDL) levels. The most commonly observed ECG change among psoriasis patients was rhythm abnormalities (17, 20.7%), followed by conduction block (6, 7.3%) and ischemic changes (5, 6.1%), with ECG changes being more prevalent in cases (33, 40.24%) than controls (25, 30.49%), but with no statistical significance.

Conclusion

The higher prevalence of dyslipidemia, particularly elevated LDL levels, highlights the need for cardiovascular risk assessment in psoriasis patients. Although ECG changes were observed, their relevance to psoriasis needs to be further studied. Overall, this study emphasizes the importance of comprehensive management strategies addressing both dermatological manifestations and associated systemic conditions to improve patient outcomes in psoriasis.

## Introduction

Psoriasis is a chronic, multisystemic, immune-mediated disease marked by T-cell-mediated inflammation leading to skin lesions and joint involvement [1,2]. The pathophysiology of psoriasis is complicated, involving an abnormal immune response with elevated cytokine levels like tumor necrosis factor alpha (TNF-alpha) and interleukin-6 [3]. These not only exacerbate cutaneous lesions but also influence systemic metabolic processes, including lipid metabolism. Dyslipidemia is a frequent complication, correlating with the severity of psoriasis and potentially enhancing the risk for atherosclerotic disease.

Cardiovascular morbidity is significantly higher in subjects affected by psoriasis, even after adjusting for traditional risk factors [4]. This emphasizes the need for proactive cardiovascular screening in psoriasis patients. Electrocardiography (ECG) serves as an essential, non-invasive tool in this context, capable of detecting early cardiac abnormalities that may indicate underlying ischemic heart disease or arrhythmic conditions [5].

Given the multisystemic nature of psoriasis and its associated risks, accurate assessment and monitoring of disease severity and response to treatment are very crucial, for which the psoriasis area and severity index (PASI) is considered the gold standard.

The association of psoriasis, lipid abnormalities, and cardiovascular disease prompts a focused examination of these parameters in affected individuals. Screening for lipid profile and ECG changes in patients with varying severities of psoriasis can facilitate early detection, enabling timely intervention to mitigate the risk of cardiovascular complications [6].

The purpose of this study was to examine the frequency and severity of lipid abnormalities and ECG changes in patients with psoriasis when compared with controls at a tertiary care hospital.

## Materials and methods

A comparative cross-sectional study was done after obtaining ethics approval from the Institutional Research Ethics Committee of the Sri Ramachandra Institute of Higher Education and Research (approval number: CPS-MED/22/JUL/77/65). A total of 164 (82 psoriasis cases and 82 controls) participants attending the outpatient department of Dermatology at Sri Ramachandra Institute of Higher Education in Chennai, India, were included in the study conducted between June 2022 and June 2024 after obtaining consent.

Patients with psoriasis above 18 years of age who were not on any medications affecting lipid metabolism for at least one month prior to participation were included in the study. Controls were patients above 18 years who came for other complaints excluding psoriasis and were not on medications affecting lipid metabolism. Cases and controls were matched for age and sex.

A detailed clinical history was obtained (including demographic details, smoking/alcohol habit, duration of disease, triggers, associated comorbidities, complications, and treatment history). Height and weight were recorded, and body mass index (weight in kilograms/height^2^) was calculated. Patients were clinically examined and categorized into mild, moderate, and severe psoriasis based on the PASI score. Venous blood samples were drawn to obtain the fasting lipid profile (enzymatic method) after an overnight fasting of about eight hours. A 12-lead electrocardiograph was recorded and interpreted with the help of a general physician. According to the National Cholesterol Education Program (NCEP) Adult Treatment Panel (ATP) III guidelines, dyslipidemia was considered when patients had any one of the following, as represented in Table 1 [7].

**Table 1:** Dyslipidemia criteria according to NCEP ATP III guidelines NCEP ATP III: National Cholesterol Education Program Adult Treatment Panel III Reference: [7]

Parameter	Threshold for dyslipidemia (according to NCEP ATP III)
Cholesterol	≥200 mg/dl
Low-density lipoprotein	≥130 mg/dl
Triglycerides	≥150 mg/dl
High-density lipoprotein	≤40 mg/dl

Statistical analysis

The obtained data were compiled using Microsoft Excel (Version 2016, Microsoft Corporation, Redmond, Washington, United States) and analyzed with the IBM SPSS Statistics for Windows, Version 27.0 (Released 2020; IBM Corp., Armonk, New York, United States). Descriptive statistics were used to summarize the data: categorical variables were presented as frequencies and percentages, while continuous variables were expressed as mean±standard deviation. The Shapiro-Wilk test was applied to assess the normality of the numerical data. Intergroup comparisons of continuous variables were conducted using the Mann-Whitney U test for non-normally distributed variables. The chi-squared or Fisher's exact test was used for comparing categorical variables across groups. A p-value of <0.05 was considered statistically significant. 

## Results

Included in the study were 82 cases and 82 controls, with a female preponderance in both cases and controls. The mean age was 45.29±13.20 years among the psoriasis group and 46.21±13.61 years among the control group. The youngest recorded age was 21 years, while the oldest was 72 years. Demographic parameters and personal habits among cases and controls are detailed in Table 2.

**Table 2:** Descriptive analysis of demographic parameters and personal habits in the psoriasis group and the control group BMI: body mass index

Parameters	Psoriasis group (N=82)	Control group (N=82)
Gender, n (%)		
Female	46 (56.1%)	46 (56.1%)
Male	36 (43.9%)	36 (43.9%)
Height (in cm), mean±SD	164.05±9.66	158.23±7.42
Weight (in kg), mean±SD	67.01±14.01	64.77±10.44
BMI category (kg/m^2^), n (%)		
<18.5, underweight	3 (3.7%)	2 (2.4%)
18.5-24.9, normal	41 (50%)	37 (45.1%)
25-29.9, overweight	31 (37.8%)	33 (40.2%)
>30, obese	7 (8.5%)	10 (12.3%)
Personal habits, n (%)		
Smoking	2 (2.4%)	11 (13.4%)
Alcohol	10 (12.2%)	18 (22%)
Smoking and alcohol	6 (7.3%)	4 (4.8%)
Nil	64 (78%)	49 (59.8%)

The most prevalent type of psoriasis was chronic plaque type (49, 59.8%), followed by palmoplantar psoriasis (20, 24.4%) and sebopsoriasis (6, 7.3%) (Table 3). The most common site of involvement was the upper limb (69, 84.1%), followed by the lower limb (66, 80.5%), the trunk (50, 61%), the scalp (38, 46.3%), the genital (13, 15.9%), and the face (9, 11%). The mean PASI was 6.89 among cases, with the highest PASI score of 28 and the lowest of 1.2. Thirty-four (41.5%) psoriasis patients had winter exacerbations of lesions, with the majority being female patients (21, 61.7%).

**Table 3:** Descriptive analysis of the types of psoriasis in the psoriasis group (N=82)

Types of psoriasis	Frequency (n)	Percentages (%)
Chronic plaque	49	59.8%
Palmoplantar psoriasis	20	24.4%
Sebopsoriasis	6	7.3%
Erythrodermic psoriasis	4	4.9%
Pustular psoriasis	2	2.4%
Guttate psoriasis	1	1.2%

Among 82 cases, 50 (61%) had nail involvement, with pitting being the most prevalent finding (Table 4). A statistically significant association was found between nail changes and severity of psoriasis (p=0.022) (Table 5). Psoriatic arthritis was diagnosed in seven psoriasis patients, with the highest PASI score of 28. No significant association was found between nail changes and psoriatic arthritis (p=0.700).

**Table 4:** Descriptive analysis of nail involvement in the psoriasis group (N=82)

Parameters	Frequency (n)	Percentages (%)
Nail involvement		
No	32	39%
Yes	50	61%
Nail changes, if yes		
Pitting	25	50%
Subungual hyperkeratosis	12	24%
Onycholysis	12	24%
Beaus line	2	4%
Leukonychia	6	12%
Chronic paronychia	3	6%
Melanonychia	2	4%
Longitudinal ridging	3	6%
Trachyonychia	2	4%

**Table 5:** Comparison of severity of psoriasis with nail involvement in the psoriasis group (N=82) *: p<0.05 considered significant

Severity of psoriasis	Nail involvement		χ^2^	df	Effect size (Cramer's V)	P-value
	No (n=32)	Yes (n=50)				
Mild	18 (56.25%)	19 (38%)	7.667	2	0.306	0.022*
Moderate	12 (37.5%)	15 (30%)				
Severe	2 (6.25%)	16 (32%)				

**Figure 1:**
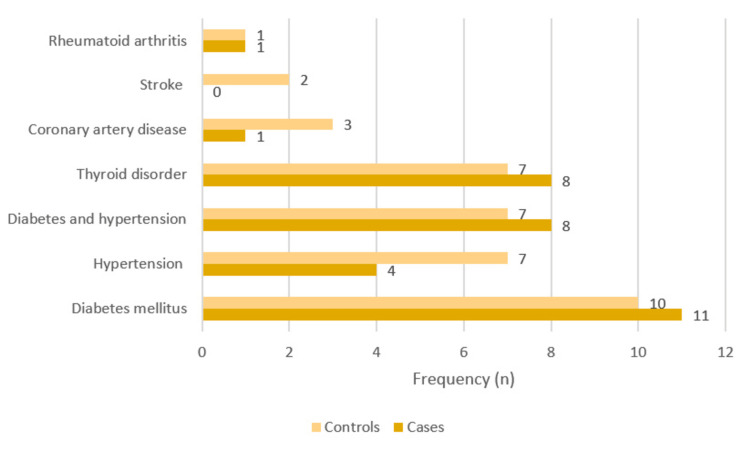
Clustered bar graph depicting the associated comorbidities in the psoriasis case and control groups

A higher prevalence of diabetes, followed by diabetes with hypertension and hypothyroidism, was seen in both the cases and controls (Figure 1).

Psoriasis and lipid parameters

Dyslipidemia was more prevalent in the psoriasis group (61, 74.3%) when compared to controls (34, 41.46%). On individual parameters, statistical significance was seen between psoriasis and low-density lipoprotein (LDL) levels (p=0.029) when compared to controls (Table 6). Statistical insignificance was found between lipid parameters and severity of psoriasis. There was no significant association found between lipid parameters and type of psoriasis, lipid parameters, and nail changes.

**Table 6:** Comparison of lipid parameters in psoriasis cases vs. controls (N=164) *: p<0.05 considered significant

Lipid parameters	Group				χ^2^	df	Effect size (Cramer's V)	P-value
	Psoriasis group (n=82)	Mean±SD	Control group (n=82)	Mean±SD				
Cholesterol								
<200	51 (62.2%)	184.2±45.4	62 (75.61%)	178.7±39.6	3.44	1	0.145	0.064
≥200	31 (37.8%)		20 (24.39%)					
Triglycerides								
<150	66 (80.49%)	119.1±69.3	61 (74.39%)	124.3±40.4	0.873	1	0.073	0.350
≥150	16 (19.51%)		21 (25.61%)					
Low-density lipoprotein								
<130	56 (68.29%)	112.0±30.8	68 (82.93%)	104.7±27.1	4.761	1	0.17	0.029*
≥130	26 (31.71%)		14 (17.07%)					
High-density lipoprotein								
<40	43 (52.44%)	40.2±7.1	32 (39.02%)	40.8±8.3	2.973	1	0.135	0.085
≥40	39 (47.56%)		50 (60.98%)					

Psoriasis and ECG changes

**Figure 2:**
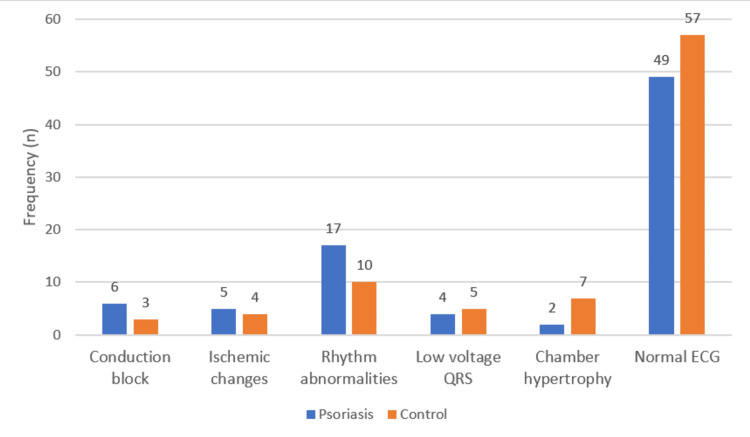
Bar graph depicting ECG changes in the psoriasis and control groups ECG: electrocardiography

Among the ECG changes, rhythm abnormalities (Figure 2) were the most common change noted in both cases (17, 20.73%) and controls (10, 12.2%), with higher prevalence in cases with no statistical significance, followed by conduction block, seen in six (7.32%) psoriasis patients and three (3.66%) of the control group, ischemic changes seen in five (6.1%) patients and four (4.88%) in controls with no statistical difference. Twenty-nine (87.8%) psoriasis patients with ECG changes had both diabetes and hypertension as comorbidities. Male patients (15, 45.6%) had a higher percentage of ECG changes when compared to their female counterparts (10, 30.3%), whereas ECG changes showed no significant differences between male and female patients in the control group.

Though ECG changes were more prevalent in the psoriasis group (33, 40.24%) than controls (25, 30.4%), no significant association was found between ECG changes and the presence of psoriasis (p>0.05), severity of psoriasis, and type of psoriasis (Table 7).

**Table 7:** Comparison of ECG changes among psoriasis cases vs. controls (N=164) ECG: electrocardiography

ECG changes	Group		χ^2^	df	Effect size (Cramer's V)	P-value
	Psoriasis group (n=82)	Control group (n=82)				
Conduction block						
Absent	76 (92.68%)	79 (96.34%)	1.058	1	0.08	0.495
Present	6 (7.32%)	3 (3.66%)				
Ischemic changes						
Absent	77 (93.9%)	78 (95.12%)	0.118	1	0.027	1.000
Present	5 (6.1%)	4 (4.88%)				
Rhythm abnormalities						
Absent	65 (79.27%)	72 (87.8%)	2.172	1	0.115	0.140
Present	17 (20.73%)	10 (12.2%)				
Low voltage QRS						
Absent	78 (95.12%)	77 (93.9%)	0.118	1	0.027	1.000
Present	4 (4.88%)	5 (6.1%)				
Chamber hypertrophy						
Absent	80 (97.56%)	75 (91.46%)	2.94	1	0.134	0.167
Present	2 (2.44%)	7 (8.54%)				
Normal ECG						
Absent	33 (40.24%)	25 (30.49%)	1.707	1	0.102	0.191
Present	49 (59.76%)	57 (69.51%)				

## Discussion

In this study, we described the demographic details, lifestyle factors of both cases and controls, and clinical features of psoriasis patients. Chronic plaque psoriasis was the most predominant pattern identified, which was consistent with patterns of multiple studies [8-11]. Patients with severe psoriasis showed significant nail involvement (p<0.022) compared to those with mild psoriasis. This emphasizes the importance of early nail examinations in patients with psoriasis who have only a few skin lesions, enabling timely intervention to prevent disease progression.

No significant association was found between psoriasis and personal habits such as smoking or alcohol consumption. This could be attributed to the predominance of female participants, where cultural constraints may have limited such associations. Furthermore, no correlation between psoriasis and body mass index was observed in our study.

This comparative cross-sectional study identified a significantly higher prevalence of dyslipidemia among psoriasis patients compared to controls (p<0.002), exceeding the rates reported in other studies [12,13]. Among the lipid parameters, atherogenic LDL was notably elevated in psoriasis patients. Additionally, 33.3% of those with dyslipidemia had comorbidities such as diabetes and hypertension. This suggests that lipid abnormalities in psoriasis may result from a synergistic interaction between environmental factors and internal metabolic dysfunction. The association between psoriasis and dyslipidemia is predicated on Th1-cell activation, autoantibodies against oxidized LDL, and the influence of cytokines (TNF-α, IL-1, and IL-6). They change the structure of lipoproteins by influencing hepatocyte function and promoting lipolysis, which causes the liver to produce new fatty acids [14]. Furthermore, dyslipidemia in this study may have been influenced by underlying conditions such as diabetes, hypertension, and other comorbidities. Therefore, baseline and regular monitoring of lipid levels is essential, with an emphasis on lifestyle modifications to prevent complications.

ECG abnormalities were more prevalent in psoriasis patients than in controls, with 87.5% of psoriasis patients with ECG changes also having hypertension and diabetes. This finding supports the hypothesis that psoriasis and cardiovascular abnormalities share common mechanisms, including genetic factors, inflammatory pathways, adipokine production, insulin resistance, altered lipoprotein function, angiogenesis, oxidative stress, and hypercoagulability [15].

Our study reported rhythm abnormalities, with sinus tachycardia being the most common ECG finding in psoriasis patients. This contrasts with the findings of Aryanian et al. [16], where atrial fibrillation was more prevalent, and Poorzand et al. [17], who reported left ventricular dysfunction as a common ECG finding. These variations highlight the diverse cardiovascular manifestations associated with psoriasis. In this study, the youngest patient with ECG abnormalities, a 32-year-old man, presented with a left bundle branch block, an important finding often associated with a previous myocardial infarction. This case underscores the need for early cardiovascular screening in psoriasis patients to detect subtle cardiac changes and prevent future complications. Additionally, ECG abnormalities were more frequent in male psoriasis patients compared to females, potentially due to the protective effects of estrogen in females, such as vasodilation, accelerated endothelial repair, and atheroprotective effects [18]. However, despite the higher prevalence of ECG changes in psoriasis patients, no significant association was found between ECG abnormalities and the disease per se, warranting further large-scale studies to explore this correlation.

Though this study highlights the importance of screening patients with psoriasis for dyslipidemia and cardiovascular changes, the abovementioned findings might have yielded a better outcome in the absence of the following constraints. Firstly, it was a single-center design, which limits the generalizability of results. Furthermore, a restricted sample size could have hampered the findings, and most significantly, multiple comorbidities could have influenced the lipid parameters and ECG changes and thus cannot establish the causal association between psoriasis and the parameters. Future research should involve multi-center, larger-scale studies to improve generalizability and control for comorbidities, enabling better assessment of causal links between psoriasis, lipid abnormalities, and ECG changes. Longitudinal studies could further clarify these associations over time.

## Conclusions

The high prevalence of dyslipidemia, particularly elevated LDL levels, highlights the need for cardiovascular risk assessment in psoriasis patients. Although ECG changes were observed to be more common in psoriasis patients, their relevance remains to be further studied. Overall, this study underscores the multidimensional nature of psoriasis and emphasizes the importance of comprehensive management strategies addressing both skin manifestations and associated systemic conditions to improve patient outcomes.
